# Sequencing artifacts in the type A influenza databases and attempts to correct them

**DOI:** 10.1111/irv.12239

**Published:** 2014-02-07

**Authors:** David L Suarez, Nikki Chester, Jason Hatfield

**Affiliations:** a Exotic and Emerging Avian Viral Disease Research Unit, Southeast Poultry Research Laboratory, Agricultural Research Service, USDAAthens, GA, USA; b Athens AcademyAthens, GA, USA

**Keywords:** databases, errors, influenza, sequence

## Abstract

**Background:**

There are over 276 000 influenza gene sequences in public databases, with the quality of the sequences determined by the contributor.

**Objective:**

As part of a high school class project, influenza sequences with possible errors were identified in the public databases based on the size of the gene being longer than expected, with the hypothesis that these sequences would have an error. Students contacted sequence submitters alerting them of the possible sequence issue(s) and requested they the suspect sequence(s) be correct as appropriate.

**Methods:**

Type A influenza viruses were screened, and gene segments longer than the accepted size were identified for further analysis. Attention was placed on sequences with additional nucleotides upstream or downstream of the highly conserved non-coding ends of the viral segments.

**Results and Conclusions:**

A total of 1081 sequences were identified that met this criterion. Three types of errors were commonly observed: non-influenza primer sequence wasn't removed from the sequence; PCR product was cloned and plasmid sequence was included in the sequence; and Taq polymerase added an adenine at the end of the PCR product. Internal insertions of nucleotide sequence were also commonly observed, but in many cases it was unclear if the sequence was correct or actually contained an error. A total of 215 sequences, or 22.8% of the suspect sequences, were corrected in the public databases in the first year of the student project. Unfortunately 138 additional sequences with possible errors were added to the databases in the second year. Additional awareness of the need for data integrity of sequences submitted to public databases is needed to fully reap the benefits of these large data sets.

## Introduction

The availability of influenza sequences in public databases has continued to expand at a rapid rate with over a quarter million accessions accessible, representing over 65 000 isolates (Influenza Research Database website http://www.fludb.org). Because of the large amount of influenza sequence data, several websites have been specifically developed to help organize, sort, and analyze the vast amount of data. The sequence that is available in the public databases is entered by the persons or research groups that generate the data, and they retain control over the sequence and can continue to make changes or corrections in the data. The data, once in the public databases, can then be used worldwide for any additional research or data analysis. There is not a minimum level of sequencing redundancy required for data that are entered into the databases, and the submitters of the data are responsible for assuring the accuracy of the data that are submitted. For direct submissions to GenBank, a quality assurance review is conducted for vector contamination, proper translation of coding regions, correct taxonomy, and correct bibliographic citations.^1^ For selected viruses, including influenza, more specific submission tools are available using a ‘virus wizard' (http://www.ncbi.nlm.nih.gov/books/NBK92943/).^2^ Several influenza-specific databases also provide an influenza-specific submission portal designed to facilitate review and annotation of influenza sequences for eventual release into the public databases.^3^ However, no process is in place to examine and correct the existing data in the public databases to assure that even obvious errors are identified and corrected.

As new and improved tools become available that allow larger datasets of sequences to be analyzed, the integrity of the sequence data becomes even more important because it becomes time-consuming and is impractical for individual researchers to identify problem sequences and remove them from their analysis. This issue is particularly manifested in the non-coding region of the influenza genome, because the routine screening by the public databases only examines the coding regions for likely translation errors. Type A influenza viruses contain eight different gene segments, and each gene segment is flanked by highly conserved 12 and 13 base pairs of non-coding sequence thought to be important in controlling gene regulation.^4^ There have not been any confirmed reports of sequence upstream or downstream of these conserved terminal sequences, and therefore, a hypothesis was developed that identified these sequences with additional nucleotides outside the conserved ends as representing a sequence error that should be corrected. This suspected non-influenza sequence could be introduced in multiple ways including from vector sequence, non-influenza sequence added to primers used to amplify the viral RNA, an additional adenine added from Taq polymerase, and poor alignment of the submitted sequence.^1^,^5^,^6^ All these mistakes in sequence should be identified, and the submitting scientists should have a quality control program to avoid these errors. However, the available data show that mistakes are commonly found in the public databases.

Through a cooperative program of an active influenza research laboratory and a local high school to facilitate real-world learning opportunities in the class room, a project was developed for the high school students to identify likely sequence mistakes, classify the most likely type of error if possible, contact the submitter of the sequence, and encourage them to correct the sequence. As part of the design, using the tools available in the Influenza Research Database, the initial screen for suspect sequence was based on size, with the hypothesis that gene sequences larger than the expected size likely had some type of error. The hypothesis was at least partially supported for the sequences identified in this project.

## Material and methods

### Sequence analysis

For each of the six influenza A internal gene segments, a sequence search was conducted using the Influenza Sequence Database (IRD) (http://www.fludb.org) to identify all gene sequences with a sequence length greater than the expected maximum segment length for that gene segment (NS>890, MA>1027, NP>1565, PA>2233, PB1>2341, and PB2>2341). The screen was conducted on two different occasions, in March 2012 and March 2013. The sequences that were added from March 2012 to March 2013 were identified by subtracting the 2012 database from the 2013 database. From the identified isolates for each gene segment, a multiple sequence alignment was performed, and each sequence was determined to have either additional sequence on the 5′, 3′, both 5′ and 3′, or additional internal sequence. Isolates with additional sequence on the 5′ or 3′ ends of the segment were evaluated for the most likely cause of the additional sequence. Three categories of common mistakes were considered. The first was the addition of non-influenza sequence added to the 5′ end of a primer that was used to increase the amplification efficiency or to facilitate downstream applications such as cloning. Because the non-influenza sequence added to the 5′ end of the primer can be any sequence, we narrowed the search to only include the primers described by Hoffmann *et al*. 6 because these primer sets appear to be the most widely used for full-length segment amplification. If 5 nucleotides matched the Hoffmann 5′ extension on the 5′, 3′ or combination of 5′ or 3′, then it was concluded that it was a primer error. Second, sequence with one additional thymidine upstream of the expected 5′ end of the sequence or one adenine (A) downstream of the 3′ of the sequence looking at the coding sense sequence, or both were considered likely the result of Taq polymerase error where Taq polymerase adds an additional A to the end of a PCR product.^2^ The third type of error was the inclusion of plasmid sequence for genes that were cloned. If the 5′ or 3′ sequence was longer than 12 base pairs in length, the additional sequence was used in a Blast analysis on the NCBI website. If the Blast analysis indicated cloning plasmid sequence or a pattern of including genes of many different species that were also cloned, it was assumed that the gene was cloned and that the plasmid sequence was not removed from the sequence submitted to GenBank. Many sequences with additional 5′ or 3′ sequence did not fit these three patterns and were reported as unknown cause of sequence error. The final category was viruses with internal issues. Because the majority of isolates included only a single nucleotide addition, often in the poly A tract in the 3′ end of the sequence, it could not be determined whether the reported sequence was correct or incorrect. These sequences were not evaluated further, and no determination was made on whether these sequences were correct or not. One class of additional internal sequence became apparent, and that was the apparent duplication or insertion of influenza sequence in the gene. These recognized duplications were reported in a separate category as well.

Examination of the original research publications can provide additional information to classify the type of error, but in a cursory review of several recently published papers, not enough detail was provided in the material and methods to determine the likely cause of the additional sequence.

### Correction efforts

As part of a college preparatory high school class project in an Evolutionary Genetics course at Athens Academy, students were provided groups of sequence with possible sequencing errors and were asked to identify the likely type of error based on the criteria listed previously. The students were asked to contact the submitter of the sequence data with the goal of having the submitter review the data, and if this sequence did have an error, prompt the submitter to send a correction to the public database where the data were originally sent. Because the GenBank record does not include email information, the student tried to look at the primary publication associated with the sequence or, in many cases, had to use different internet sources to identify an active email for one of the scientists associated with the submission. Two different Athens Academy classes, the 2011–2012 and 2012–2013 classes, participated in the project. Because not all the submitters of the suspect sequences were contacted the first year, additional suspect sequences were added in 2012–2013, and many suspect sequences were not corrected after the initial contact, so the second year's class had ample sequences to evaluate or re-evaluate.

## Results

### Analysis of sequences

The hypothesis of the research was that sequences for the internal proteins found in the public databases, as accessed through the IRD website, that were over the expected maximum lengths had errors in sequencing or assembly that were not corrected before being submitted to the public databases. For the sequences that had additional nucleotides on the 5′ or 3′ ends of the sequence, the results of our analysis support the original hypothesis. We are unaware of any published cases where additional sequence was legitimately found in the sequence. However, many of the flagged sequences had insertions of sequence internally. Several examples can be cited where the gene segments are legitimately larger than the expected average, and therefore, the original hypothesis is not supported for these cases. The first clear exception is that of the non-structural (NS) gene from H17N10 bat isolates at 895 base pairs in length. The bat NS gene sequences have the conserved ends of type A influenza viruses, but also additional internal sequence.^7^ Also in several different examples, it was shown that insertion of sequence, often a single nucleotide in the non-coding region, can occasionally be observed in different gene segments, which is sufficiently supported through robust gene sequencing that included circularization of the RNA gene segments to facilitate sequencing of the non-coding region^8^–^11^(data not shown). One particular gene segment, the nucleoprotein, had a disproportionally high number of insertions in the 3′ end of the non-coding sequence (Table1).

**Table 1:** Identification of GenBank Accessions with likely sequence errors

Total	PB2	PB1	PA	NP	MA	NS	Total
Total Number of GenBank Accessions	18 372	18 394	21 813	22 132	28 776	23 037	132 524
Number Accessions above Consensus Size	162	114	234	321	104	146	1081
Additional sequence on 5′ end	28	37	22	14	28	12	141
Additional sequence on 3′ end	64	44	120	38	33	76	375
Additional sequence on 5′ and 3′ end	67	23	56	34	37	48	265
Internal insertions	3	10	36	234	6	10	299
% of errors/Total sequences in database	0.88%	0.62%	1.07%	1.45%	0.36%	0.63%	0.82%
Predicted types of errors							
Non-flu sequence related to cloning vector	30	15	31	12	18	16	122
Non-flu sequence related to Hoffmann primers	35	20	113	25	18	69	280
Taq polymerase addition of Adenine	17	17	13	15	10	9	81
Unknown	74	48	36	34	48	37	277
Internal	3	10	36	234	6	10	299
Bat, duplication	3	4	5	1	4	5	22
Total	162	114	234	321	104	146	1081

For the sequences with additional sequence upstream or downstream of the traditional ends of the RNA segment, 3 types of errors were commonly found, including non-influenza sequence added to the 5′ end of the primers used for amplification, the addition of adenine or thymidine added through the use of Taq polymerase, and the addition of plasmid sequence from genes that were cloned and then sequenced. Additionally a small number of sequences had evidence of duplication of influenza gene sequence or insertion of part of another influenza gene into the sequence. It is assumed, but it cannot be conclusively determined that this is an error in contig alignment. Finally, 25% of sequences did not have enough additional sequence to make a reasonable assumption as to the source of the error, and these sequences were left in an unknown category. A total of 1081 sequences were identified in this study as meeting the criteria of exceeding the length requirement, which represents 0.82% of the IRD database as of March 15, 2013, when the data were extracted for analysis (Table1). For the individual genes, the matrix gene had the fewest identified errors at 0.36% and the nucleoprotein had the most at 1.45%. The nucleoprotein, as mentioned previously, had 73% of suspect sequences as having additional internal sequences, and if these were excluded, it had a relatively low number of errors. Overall, the most common error observed was primer error related to the Hoffmann *et al*.^6^ paper with 25.6% (Table1). Both the NS and PA genes had over 47% of suspect sequences identified with this type of error. This second most common error was non-influenza sequence related to plasmid sequence with 11.3%, and the least common error was the addition of adenine or thymidine sequence from a Taq-related addition with 7.5%.

Excluding the gene segments with additional sequence internally, the sequences were assigned to students and they first analyzed the sequence to identify the type of error. They then attempted to find a working email for one of the submitters of the sequence. The students were encouraged to use multiple sources, starting with the National Center for Biotechnology Information's PubMed, to examine the published paper associated with the sequence. However, many of the sequences were not associated with a published paper or the email was no longer current from the published paper. In these cases, a more general internet search using the submitter's data was attempted. Students were guided in drafting an email to alert the submitter of the possibility of an error in their sequence and what type of error it likely was. There was a range of responses to these emails. Many submitters expressed appreciation for being made aware of the issue, and most who responded positively corrected their sequences quickly. Some responses argued that their sequences were not incorrect, and some specifically felt that because the sequences were accepted by the public database, they were correct sequences. A couple of submitters, after several emails of additional explanation, agreed that there was a problem and agreed to fix the sequences. However, most commonly there was no response. It could not be determined whether the non-responses were because the email addresses were no longer active or whether the submitter did not agree with the email or otherwise just decided not to respond.

Because the contacting of the submitters occurred in two different year's classes, the correction rate was calculated after the first year and after the second year. A number of additional suspect sequences, a 14% increase, were also added between the first analysis and the second analysis (Table2). In the first analysis, 943 suspect sequences were identified, and 215 or 22.8% of the sequences were eventually corrected in the public databases. When the databases were queried on March 15, 2013, an additional 138 suspect sequences were added to the public databases. As of August 15, 2013, an additional 19 sequences were corrected for a total of 234 sequences or 21.7%.

**Table 2:** Total number of sequences corrected as part of the project

	PB2	PB1	PA	NP	MA	NS	Total
Corrected sequences on March 19, 2013	69	18	102	13	3	10	215
Uncorrected sequences March 19, 2013	82	85	118	252	94	97	728
% Corrected March 19, 2013	45.70%	17.48%	46.36%	4.91%	3.09%	9.35%	22.80%
Additional Sequences added March 2012–March 2013	11	11	14	56	7	39	138
Total Corrected sequences August 15, 2013	71	19	106	15	9	14	234
% Total corrected	43.83%	16.67%	45.30%	4.67%	8.65%	9.59%	21.65%
Total uncorrected	91	95	128	306	95	132	847

## Discussion

Through a science, technology, engineering, and mathematics (STEM) outreach program, a collaborative project was developed between an active influenza laboratory and a local high school class studying Evolutionary Genetics. The goal of the project was to give students an active learning experience where their efforts could actually have a meaningful impact on the science community. The students had to learn how to use common bioinformatics tools including Blast analysis, multiple sequence alignments, phylogenetic trees, and work with large databases. The Influenza Research Database was selected as the primary public database because of the ability to have private and shared workbenches that facilitated data sharing. Other websites also needed to be accessed, including GenBank and PubMed, in an effort to find submitter's emails. Overall the project was successful with almost 22% of the suspect sequences having been corrected, and the percentage corrected is 30% when the internal gene segments were eliminated because submitters were not contacted for those samples.

Four specific types of errors were identified for gene segments with additional sequence upstream or downstream of the conserved ends. The most common error observed could be traced to a single published paper which describes the full-genome amplification of influenza gene segments by Hoffmann *et al*.^6^ This article has been referenced at least 676 times in publications according to a recent SCOPUS search. The procedure uses primers targeted separately to all gene segments using the highly conserved 12 and 13 base pair sequences on the end of all eight gene segments and an additional 2–8 base pairs specific for that gene segment. To improve the PCR amplification efficiency, because many of the primers used are relatively short and have low annealing temperatures, 5′ extensions of 15 base pairs of non-influenza sequence containing a restriction enzyme site compatible with reverse genetics plasmids were included in each primer. Although it is considered good laboratory practice after sequencing to remove the primer sequence before submission to a public database, this step was not implicitly included in this article. Our analysis of the public database shows that a number of influenza sequences failed to remove the Hoffmann primers from their submitted sequence based on the unique non-influenza sequence that was added to the ends of these primers. It was presumed that if at least 5 nucleotides of the non-influenza part of the Hoffmann primer were adjacent to the 12 and 13 conserved ends of the influenza sequence submitted to GenBank, then likely the sequence was generated by amplification with the Hoffmann primers. Additional sequences likely contained the non-influenza Hoffmann-related sequence, but the submitted sequence was less than 5 bp or the non-influenza sequence was on only one end of the gene segment and the overall length was equal to or less than the expected size. Additionally, many scientists have developed their own amplification primers with unique 5′ extensions, and some of these suspect sequences could only be confirmed when reading the published paper that accompanied the sequence. However, many research papers no longer provide primer information used for PCR amplification or sequencing, so it was not possible to confirm the specific cause of the additional sequence. It is likely that many of the “unknown” samples were primer related, and the submitter failed to properly trim the sequences before submitting to one of the public databases.

The second most common problem was the inclusion of vector sequence in the submitted data. The public databases do have screening programs to identify and prompt submitters to remove vector data.^1^ However, these programs also require a minimum length to be able to match their database. For our analysis, at least 12 contiguous base pairs were required to perform the Blast analysis that was needed to identify a likely plasmid sequence. The third most common error was related to Taq polymerase. Taq polymerase, as well as other polymerases, is known to add a single adenine to the end of the PCR product as part of the amplification process.^5^ This additional sequence is non-templated DNA and should be trimmed along with the primer sequence, but in at least in 81 cases, it was not. The final identified mistake was the presence of duplicated or inserted influenza DNA on the end of a sequence. In every case, the additional sequence was on the 3′ end of the positive coding sense gene segment. The sequence could be from the same gene segment, but more commonly it was sequence from another gene segment. It was presumed that this sequence was improperly aligned and the sequence was incorrect, because no published paper explained that the duplication was likely correct.

The original hypothesis was that any sequence larger than the expected size was likely a sequence error. This hypothesis is largely supported, although a few exceptions were observed for those sequences with additional sequence internally. Several laboratories were identified that had repeatedly made the same error, and in general, these laboratories were receptive in correcting their sequence. However, a large number of laboratories were identified worldwide with some type of error. The non-US laboratories provided the most difficulty in getting a viable email and/or response to the email question. It is possible that the laboratories did not receive the email or had difficulty understanding the email because of language issues.

This project identified only a small percentage of errors in the influenza database. However, the true number of errors is likely much higher for three reasons. First, in an attempt to simplify the procedures for the high school students, it was decided that only the internal genes would be examined in this study and that the minimum size screening would be the primary screening method. The influenza internal genes are remarkably similar in size with only a relatively small number of confirmed size variants, and therefore, screening based on the minimum segment length would identify a large number of sequences that could be analyzed. However, the hemagglutinin and neuraminidase genes naturally vary in size between subtypes and even within subtypes, and it would require different screening methods than that used for the internal genes. Preliminary analysis (data not shown) not surprisingly shows similar types of errors in the hemagglutinin and neuraminidase genes. Second, if an error is only on one end of a gene segment and there is incomplete sequence on the other end of the gene segment, then the gene segment may not cross the size threshold used as the initial filtering step for inclusion in the project. This type of error would require different screening methods other than by size. One alternative is to use Blast analysis when searching for particular sequences such as the Hoffmann amplification primers. Using these methods, additional suspect sequences were identified (data not shown). The third type of error that was not specifically classified in this study is based on the inclusion of primer sequence in what is submitted to the sequencing databases that does not match the viral template. Although the primer sequence needs to closely match the target sequence to amplify the viral RNA, the amplification can still occur if there are one or two mismatches in primer and target especially if the mismatches are on the 5′ end of the primer. Because the PCR product will reflect the primer sequence and not necessarily the target sequence at the primer sites, it is good laboratory procedure to remove the primer sequence from what is submitted to the public databases. However, this procedure is not uniformly practiced based on the results of this research. In addition, it is likely that laboratories correctly removed the non-influenza sequence, but failed to remove the primer sequence, particularly for the non-coding sequence. The attitude of some laboratories is that the only important part of the sequence is the coding sequence, and less attention is given to the non-coding sequence. The popularity of the Hoffmann primers is that the entire coding sequence is included and the entire gene can be cloned for use with reverse genetics or other downstream applications. The primer sequences are in highly conserved regions and therefore present a low likelihood of introducing errors through the sequencing process. However, few laboratories routinely do the extra step to actually sequence the entire non-coding region because the extra time and expense needed. The most common method for sequencing the non-coding region is to circularize the RNA gene segment using T4 RNA ligase and then RT-PCR amplify across the ligated RNA to amplify the non-coding region.^8^–^11^, essentially twice the work for little additional sequence, but a necessary step to get correct full-length sequence.

This study highlights the problems of data integrity of the influenza database, although the same problems likely occur for other sequence datasets as well. The public databases, such as GenBank, ultimately rely on the submitter for assurance of data integrity of what is submitted. Errors, however, are routinely introduced by even experienced laboratories into the public databases. Correcting errors in GenBank is a relatively easy process that requires one of the original submitters to send an email directly to GenBank with the revised or corrected sequence. The GenBank staff reviews the changes and typically corrects the accession in a few days. The original data can still be accessed using the unique accession number, so it does provide a permanent record of the original submission, but only the updated sequence is accessible for search or Blast analysis. However, there is no mechanism for third parties to directly correct or flag suspect sequences in the public databases. The Influenza Research Database website does provide a notation of sequences with likely errors on their site, but this additional annotation is for informational purposes only. 3

Scientists are profiting from a huge increase in data and new tools to analyze the data, but clearly the strength of the analysis is only as good as the quality of the data that are available. In a comparison of the available non-coding sequences, it is clear that the obvious mistakes and the not so obvious mistakes are increasing the difficulty to understand the data and are likely contributing to bias in the data that are leading to false conclusions. This high school class project will continue to identify and work to correct both the current mistakes and the future mistakes that will be introduced into the public databases as both a learning experience and for the public good. As the instructors improve their teaching methods allowing new bioinformatics tools to become available, the students will be able to identify more and different types of errors as part of their learning experience and hopefully excite them about careers in STEM fields to foster the next generation of scientists.
